# HOLD study (Home care Obstructive Lung Disease): natural history of patients with advanced COPD

**DOI:** 10.1186/s12904-016-0104-9

**Published:** 2016-03-22

**Authors:** Daniel Gainza Miranda, Eva María Sanz Peces, Alberto Alonso Babarro, Maria Concepción Prados Sánchez, María Varela Cerdeira

**Affiliations:** ESAPD Dirección Asistencial Norte de Madrid. C.S. Reyes Católicos, Avenida de España 20, San Sebastian de los Reyes, Madrid, Spain; Unidad de Cuidados Paliativos del Hospital de la Paz, Paseo de la Castellana, 261, 28046 Madrid, Spain; Servicio Neumología del Hospital de la Paz, Paseo de la Castellana, 261, 28046 Madrid, Spain; Equipo Soporte Hospitalario Hospital Ramon y Cajal, Ctra. de Colmenar Viejo, km. 9,100, 28034 Madrid, Spain

**Keywords:** COPD, Palliative care, Homecare, End of life

## Abstract

**Background:**

Chronic obstructive pulmonary disease (COPD) is the fourth cause of death in western countries. Its final stage has clearly been forgotten by medical research in recent years. There exists consensus regarding the need to integrate palliative care in assisting these patients, but the difficulty in establishing a prognosis for the disease, establishing limits for life support measures, the lack of information about the disease’s natural course and ignorance as to the most effective health-care structure for these patients’ palliative treatment may be responsible for their late inclusion or non-inclusion in specific programmes. The main purpose of this work is to find out the natural background of patients with stage IV COPD and the main prognostic factors that influence these patients’ survival.

**Methods/design:**

Prospective observational study of a home patient cohort with stage IV COPD sent from Neumology consultations and Palliative Care Unit in La Paz Hospital in Madrid and Primary Care Health Centres in the area to the palliative care home support team. The goal is to study socio-demographic variables, prognosis, nutritional status, use of health resources, perceived quality of life, functionality, main symptomatology, use and effectiveness of opioids, adherence to treatment, prognostic information regarding the disease, information given by professionals, advance directives, social backup requirements and overburden level of the main caregiver.

**Discussion:**

The HOLD study is a project aimed at finding out the prognostic factors and evolution of the disease COPD in its most advanced stage. The final goal is to improve the health and quality of life, in a personalised, integral way up to end of life and explore and foster communication with patients, as well as their participation and collaboration in decision-taking. The HOLD study can help us better understand what these patients’ real palliative and care needs are, in order to more efficiently organise their treatment at end of life.

## Background

Chronic Obstructive Pulmonary Disease (COPD) is currently the fourth cause of death in western countries [1, 2], and it is, among the main ones, the one that has most increased in recent years [1]. The advanced stages of the disease are characterised by significant morbidity. The high frequency of symptoms, functional deterioration and the large number of exacerbations lead to major deterioration in patients’ quality of life, to a high rate of hospitalisations and, in general, to a significant increase in care and treatment costs [3, 4].

The final stage of progressive, incurable diseases have been ignored by medical research until recent years. In general, patients in more advanced states have been excluded from research work. However, only a proper description of the final stage of these diseases can let us better evaluate patients’ needs and the care and treatment they should receive [5]. The results of the SUPPORT study [6] showed the significant deficiencies in the care of these ill people which comprise both the treatment of symptoms and the consideration of patients’ advance directives.

There is consensus regarding the need to integrate palliative care in attending to patients with COPD. In recent years, practically all scientific organisations that include professionals involved in attending these patients and different health-care institutions have made major efforts to try to divulge the need to apply the principles of palliative care to these patients [7–11]. The main problem in implementing palliative care programmes for patients with advanced COPD lies in establishing appropriate prognostic criteria. Some prognostic indexes have been created to help in selection processes. The BODE index [12] is the most widespread [13, 14]. However, even patients with very high values on the BODE index may survive for years. The patient cohort with Advanced Chronic Respiratory Disease in the SUPPORT study [15] showed how patients with worse self-perception of their state of health survived less. Subsequently, other works have shown how ratings obtained by questionnaires on health-related quality of life, both general and specific, are correlated with survival [16, 17]. The difficulty in estimating the survival of patients with advanced COPD may mean that they receive sub-optimal treatment.

Despite showing a comparable frequency of symptoms and, in general, similar needs, when end-of-life care received by the patients with lung cancer is compared with that of advanced COPD sufferers, favourable results are always obtained for oncological patients [18]. In these studies, patients with advanced COPD show: worse health and social care, worse diagnostic and prognostic information, worse symptomatological control, less participation in clinical decision-taking and greater use of aggressive measures to increase survival [15, 19–21]. In a study carried out by Heyland *and cols*. [22], patients with advanced-stage COPD reported, as high-priority needs, good symptomatic control, not being a burden for their family, receiving appropriate information which includes benefits and risks of the treatment, and having access to a doctor to ask questions regarding their disease.

In short, the difficulty in establish a prognosis, the difficulty in limiting advanced life-support measures and the lack of information about the disease’s natural course can be responsible for the these patients’ late inclusion or exclusion in palliative care programmes. In recent years, authors have recommended, in order to improve this situation, including palliative care as part of the standard team responsible for monitoring patients in advanced stages, in such a way that a palliative approach to the disease starts early, regardless of their prognosis [23].

Continuity of care is a key element in offering proper health care to patients with advanced diseases [24]. A single team, especially if it is hospital-based, cannot respond to patient’s needs under any circumstance. For this reason, coordination is essential with different care resources. Home care allows attention to be given directly to advanced-COPD patients [25] whose functional deterioration prevents them from leaving home and attending consultations. It would therefore be a key factor for ensuring continuity of care.

There barely exist works on the most effective health structure for attending to end-of-life for these patients [24], nor on the natural background of patients with advanced COPD. These kinds of studies can provide us with information about symptomatic burden, quality of life and real needs of health resources for these patients. This information would let us develop more effective health structures and improve the care that these patients receive, offering them and their families more realistic expectations as the disease progresses and they approach the moment of death.

The main goal of the study is to find out the natural background of stage-IV COPD patients, from when the advanced disease obliges them to stay at home, prospectively evaluating the evolution of symptoms, quality of life and the use of health resources in the final stage of the illness.

The specific goals of this work are:

1. Find out the main prognostic parameters in these patients survival and in the evolution of their quality of life
2. Assess the level of information to patients with COPD and to their families, the percentage of patients with advance directive documents and the level of information given by professionals and included in their reports
3. Find out the evolution of COPD patients functionality, describe the most important symptoms, paying special attention to dyspnoea, as well as its level of control and co-morbidity
4. Evaluate use of health resources in advanced stages of COPD patients monitored at home
5. Evaluate perceived quality of life and its evolution in the advanced stage of the COPD at home
6. Estimate the use of opioids in standard clinical practice for treating dyspnoea and their effectiveness in controlling dyspnoea
7. Assess the level of therapeutic compliance and inhalant techniques and the use of home oxygen therapy
8. Find out the main caregiver’s social support needs and level of over-burden

## Methods

### Design

Prospective observational study of a stage-IV COPD patient cohort immobilised at home.

### Participants and recruitment

The study will include patients sent to the Palliative Home Care Support Team (PHCST) from the Neumology Service in La Paz Hospital, from the Palliative Care Unit, or from Primary Health Care diagnosed with stage IV COPD who are going to be monitored at home and who fit the criteria for inclusion, exclusion and withdrawal (Table 1).

**Table 1:** Criteria for selecting patients

Criteria for inclusion	Criteria for exclusion	Criteria for withdrawal
• Over 18 years old • Clinical diagnosis of stage-IV COPD according to GOLD 2007 guidelines • Functional deterioration that hampers monitoring in external consultations or PPS below 70	• Severe cognitive deterioration or severe mental illness • Diagnosed with lung cancer or cystic fibrosis • Impossibility of maintaining home care due to absence of main caregiver • Not fluent in Spanish language	• Suffering from lung cancer, of recent appearance during monitoring • Change of habitual residence outside the Northern Community of Madrid Care Directorate • Admission to a Palliative Care Unit • Patient decides to abandon the study

### Data collection

The information will be collected during programmed visits to the patients’ home, by PHCST researchers.

**Fig. 1:**
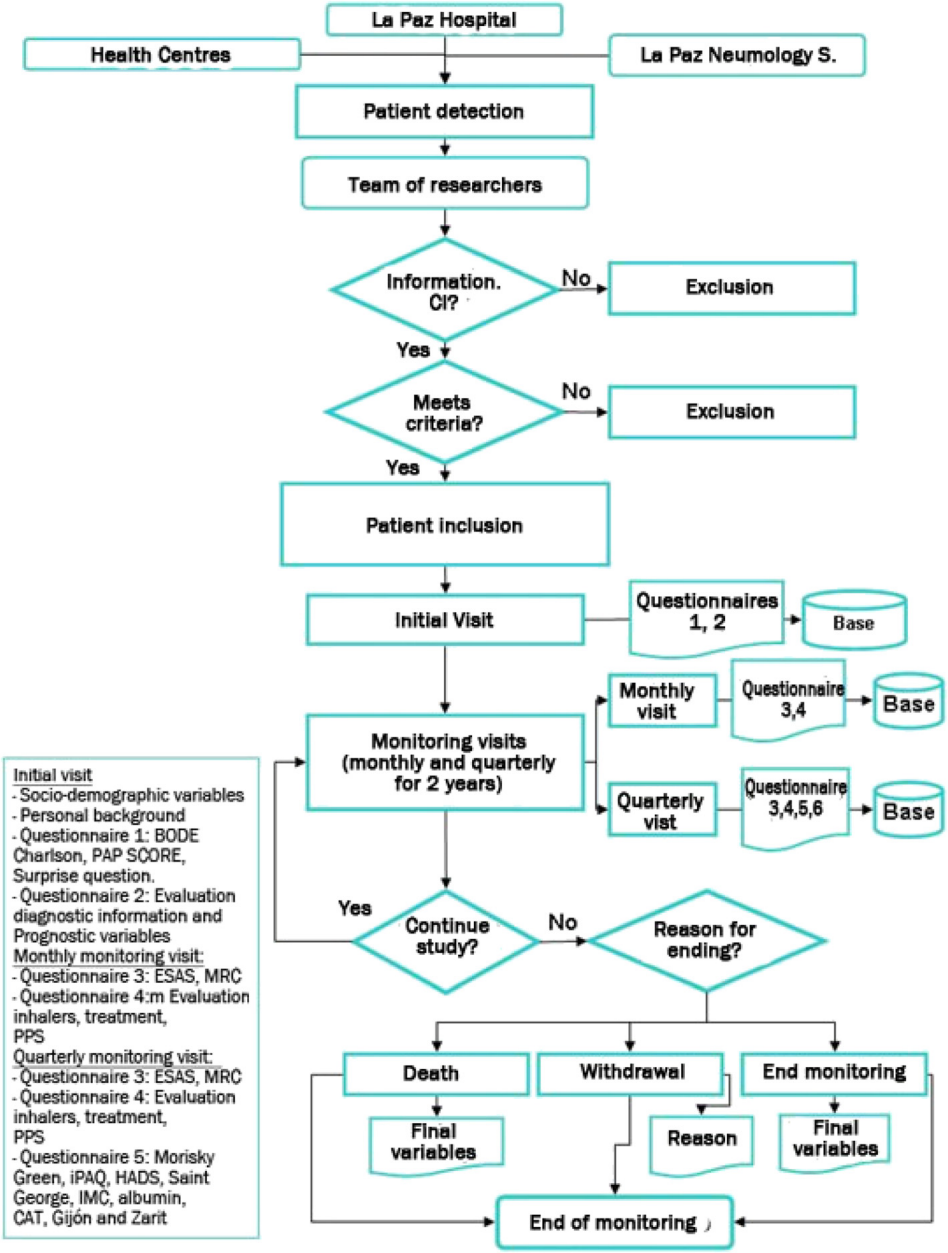
Shows the general outline of the study’s data collection

In the initial visit, apart from checking that the patient complies with the criteria for inclusion and exclusion, and the signature of informed consent, information will be gathered on the patient’s variables (listed below). Monitoring visits will be carried out quarterly up to a 2-year maximum (Fig. 1).

### Variables

During the different programmed home visits, the following variables will be collected by the research team:

#### Socio-demographic variables

Age, sex, education level, labour situation, family unit, relationship with main caregiver.

#### Survival variables

- Survival time: this will be calculated as from the registration date of the disease’s stage IV diagnosis and date of death. Survival will also be calculated as from the date of inclusion in the study.
- Place of death (Home/Palliative Care Unit/Hospital Ward/Residence/Emergency Room).

#### Personal background

Smoking, packets/year, age when smoking started, cardiac insufficiency, high blood pressure, diabetes, depression, anxiety disorder, flu and pneumococcal vaccine, Pseudomona aerogynose isolation.

#### Prognostic variables

- Charlson comorbidity index [26]: absence of comorbidity is considered: 0–1 points; low comorbidity: 2 points; and high: > 3 points. Since monitoring will be less than 5 years this will not be corrected by age.
- BODE Index [12]: calculations will collect indexes of body mass, last FEV1 upon discharge, Modified Medical Research Council dyspnoea scale and the 6-min walk distance at the end of consultations. Scored from 0 to 10 points.
- Surprise question: formulated as “would you be surprised if this patient dies the next year? YES/NO”. The care team will make this question after the patient’s first home evaluation [27].
- Palliative Prognostic Score: prognostic scale that divides patients into three risk groups: A (>70 % probabilities of survival at 30 days) if the score is between 0–5.5; B (30-70 % survival at 30 days) if the score is between 5.6 and 11; and C (probability less than 30 % at 30 days) if the score is between 11.1 and 17.5 [28].
- Nutritional state: this will be obtained via three variables. The body mass index, albumin obtained by blood analysis and Mini Nutritional Assessment (MNA): a nutritional screening and assessment instrument for the elder population [29], validated in Spanish [30].

#### Variables to determine the use of health resources and social support needs in the final stage

- Telephone calls: Made to the PHCST, Primary care health team (PCHT), emergency calls, and tele-assistance. Caregivers will be asked about calls made since the previous visit.
- Home visits: Carried out by the PHCTS (collected by care/research team),
- Visits made by primary care doctor in homes and health centres (asking the patient and/or carer and checking in the computer programme AP-Madrid visits registered by PCHT.
- Hospitalisations/hospital emergencies: collected by reports of admissions by hospital services. This will also register the number of days hospitalised.
- Admissions to Palliative Care Units/Residences: this will register the number of days admitted up to death. It will be recorded by telephoning the centre.

#### Variables to “Evaluate perceived quality of life”

- St. George’s questionnaire regarding health-related quality of life, validated in Spanish [31]: This will be checked after being carried out to evaluate its full implementation. Score variations of 4 points are considered clinically significant.
- Questionnaire regarding health-related quality of life: COPD Assessment Test (CAT) [32]. Score from 0 to 40. It divides patients into low-impact levels (<10 points), medium-impact (10 to 20 points), high-impact (more than 20 points) or very high (more than 30 points). Self-fulfilment will be supervised by the health team. Variations of 2 points in the score are considered clinically significant.

#### Variables to “Evaluate the functionality of COPD patients”

- Palliative Performance Scale (PPS) [33]: Functional assessment scale based on the Karnofsky scale. It scores from 0 to 100 % where 0 % is the patient’s death and 100 % means a healthy patient able to walk around.
- Barthel index (BI) [34]: The BI is an ordinal scale used to measure performance in activities of daily living (ADL). It aims to assign each patient a score of their level of dependence to carry out a series of basic activities. It uses ten variables describing ADL and mobility. The values assigned to each activity depend on the time used to carry them out and need for help to do so [8, 10].
- International Physical Activity Questionnaire, short version [35]: This provides information about time used when walking, activities of moderate and vigorous intensity and in sedentary activities. Physical activity is described in Metabolic Equivalent Tasks (METs), where an equivalent metabolic task is equal to the energy expended while resting, and are expressed in METs minutes/week. The IPAQ considers moderate-intensity activities to be those of 3–6 METs and vigorous to those greater than 6 METs. The questionnaire also classifies physical activity in three levels: lowers, moderate and high. Finally, the questionnaire includes an additional indicator of sedentary activity that does not add to the score, and which corresponds to time sitting down, measured separately for working days and weekends.

A drop in functionality will be evaluated by unit of time in order to evaluate the linearity of the loss and its relation with survival

#### Variables to “evaluate the most important symptoms, level of control and co-morbidity”

- Edmonton Symptom Assessment System (ESAS) in its validated Spanish version [36]: list of 10 numerical scales from 1 to 10, which evaluate the average intensity of 10 symptoms at a certain time. The patient is requested to indicate the number that best represents the intensity of each symptom. If the patient were unable to indicate it correctly, he or she will be asked about each symptom and to qualify it as zero, slight, moderate, severe or very severe, assigning for each of these answers a score of: 0 nothing, 2 for slight, 5 for moderate, 7 severe and 10 very severe.
- Dyspnoea Scale of the British Medical Research Council (MRC) [37]: this evaluates the level of dyspnoea from 0 to 4 (level 0: the dyspnoea only begins with maximum effort; up to level 4: the dyspnoea exists while resting and prevents the patient from leaving his or her home). This will be evaluated by asking the patient during a clinical interview.
- The Hospital, Anxiety and Depression Scale [38]: This consists of 14 items. It measures mood with two scales, one for anxiety and another for depression. According to the score obtained it is divided into non-case of anxiety/depression (0–7 points), doubtful case of anxiety/depression [8–10] and case of anxiety/depression if a score above 11 points is obtained.

Number of exacerbations: this will register the number of exacerbations attended during the patient’s monitoring by the PHCST and exacerbations that have required hospitalisation will be noted.

#### Variables to “Evaluate the use of opiates in standard clinical practice to treat dyspnoea and their effectiveness in controlling dyspnoea”

- Use of opioids: use yes/no, date prescribed, professional who prescribes their use.
- Effectiveness: The use of opioids will be considered effective in controlling dyspnoea if it decreases in the ESAS by at least 2 points with the response remaining for at least 3 days. A check-up will be carried out on the third day of the opiate’s prescription to ask about the dyspnoea. Time of treatment with opioids will also be evaluated, including date of treatment withdrawal and reason for doing so.

#### Variables to “Evaluate the level of therapeutic compliance and the inhalant technique and home use of oxygen therapy”

- Morinsky-Green Questionnaire validated in Spanish [39]: This evaluates patients’ attitudes regarding the treatment. Patients who respond correctly to the four questions it comprised will be qualified as compliant. An inadequate response qualifies the patient as non-compliant.
- Inhaler type and inhalant technical: This will register the type of inhalers that the patient uses and the inhalant technique will be evaluated, classifying it as correct or incorrect according to the recommendations of the Spanish Neumology and Thoracic Surgery Society (SEPAR), for the use of inhalers [38].
- Variables relating to oxygen therapy: This will record the number of hours of oxygen therapy the patient has used the previous day, the number of litres per minute determined from neumology and the number of litres per minute administered by the patient’s concentrator, checking the flow meter during the visit. It will also evaluate the increase in oxygen requirements by time unit with the aim of evaluating reality of loss and its relationship with survival. Use of non-invasive ventilation mechanics and number of hours of ventilation will be also evaluated.

#### Variables to “Evaluate the level of information to COPD patients and their families”

- Information given by the professional: This will register the patient’s reports or record. The patient will be considered as:
  Informed by the professional of the diagnosis: if the reports show that the patient has been explained that he/she suffers from COPD and its level of severity.
  Informed by the professional of the prognosis: if the reports show that the limitation of life expectancy has been explained, conditioned by the base disease.
  Partially informed: If there exists registered information as regards diagnosis and prognosis but it is incomplete (understanding complete to mean as described in the previous point).
  Not informed: if it is shown that information has not been passed on to the patient as regards the diagnosis or prognosis. Apart from background, this will also register or by contacting the responsible professional the reason for non-information (not requirement of information from the patient, difficulties in professional/patient communication, pact of silence, other).
  Not referred: the patient’s clinical record or reports do not register whether information has been given to the patient regarding the diagnosis and/or prognosis.
- Information perceived by the patient: During the clinical interview, the patient’s knowledge will be evaluated as regards their diagnosis and prognosis, classifying the patient as:
  Expert: We will consider the patient to be an expert of the diagnosis if on being asked which disease they are suffering, the patient refers to: “COPD”, “chronic bronchitis”, “bronchitis”, “lung disease”, “bronchial disease”, “chronic lung disease”. They must also express its chronic, irreversible nature: “It is chronic”, “it is incurable”, “it is irreversible”, “it is forever”, “it’s for life”, “it has no solution”. We will also consider the patient to be an expert on the prognosis if they consider that their life expectancy is limited as a consequence of their disease.
  Partially expert: During the interview, the patient does not know their diagnosis or prognosis or they partially know the information as regards diagnosis or prognosis.
  Non-expert: The patient does not know either the diagnosis or the prognosis.
  Not explored: Not approached during the interview by the team. The reason will be given (information not required by the patient, difficulties in the professional/patient communication, pact of silence, other).
- Advance directive document: The patient or his/her family will be asked as to the existence of this document, classified in the following categories:
  Non- existence: document of advance directives does not exist in writing.
  Unregistered legal document: legal document of advance directives exists but it has not been deposited in any official register of advance directives.
  Registered legal document: legal document of advance directives exists in an official register of advance directives.
  Document prepared with the team: the document of advance directives in writing has been prepared and included in the patient's clinical record or in a separate document during our team’s monitoring and with our advice. The category prepared with the Team is not exclusive from unregistered or registered legal document.

#### Variables to “Evaluate the main carer’s social support needs and level of over-burden” registering the following variables

- Gijón abbreviated and modified scale of socio-family evaluation [40]: This evaluates 3 items: family situation, relationships and social contacts and social network support.
- Zarit Burden inteview short version [41]: questionnaire of 7 items where the caregiver assigns a frequency corresponding to a score of 1 to 5. If the sum of the scores is greater than or equal to 17 it would indicate caregiver overburden.

### Calculation of sample size and data analysis

It is estimated that in the Community of Madrid (the population is around 6,400,000 inhabitants)., every year 500 COPD patients enter the final stage of the disease. Coverage by the team of professional researchers is for a population of around 1,000,000, so it is estimated that at most up to 77 patients/year could be included and with a forecast to include approximately 70 % of these patients from the area covered by the ESAPD and a 2-year period of recruitment. 108 patients would be included, which would guarantee adequate representation of this population. It is calculated that losses could mean 10-20 % of these patients, so the final sample size is considered to be between 80 and 100 patients.

The information included in the data-collection book will be recorded in a local database. This database will have the necessary protection measures and it will only be accessible by the project researchers. Prior to the statistical analysis, an analysis of the quality of the data collected will be carried out to purge any possible transcription errors.

### Statistical analysis

Most of the goals will be met with descriptive statistics, summarising the quantitative variables by means of average, standard deviation and minimum and maximum values. For asymmetric distributions, the median will be used as a centralisation measure and percentiles 25 and 75 as measures of dispersion. For categorical variables, their absolute frequency and distribution of frequencies will be expressed. Evaluation of survival will be carried out by taking into account the date when home monitoring began and date of death (or departure from the study), using a Cox proportional hazards model to examine the independent effect of the prognostic variables studied.

The statistical programme SPSS version 21.0 will be used.

## Discussion

The HOLD study is a project focused on finding out the factors prognostic and the evolution of the disease COPD in its most advanced stage. In recent years, other works have described the advanced stage of other organic insufficiencies and neuro-degenerative diseases [42, 43]. We need to have the best scientific evidence to respond to the needs of patients suffering from advanced diseases [5]. The final goal should be to improve health and quality of life in a personalised, integral way until end of life. Respiratory diseases represent the second cause as regards the type of cases attended in hospitals, accumulating 11.6 % of discharges (only behind those of the circulatory system), they are the fourth cause of death and are also among the first four diagnoses in global costs for the Spanish health system, representing the largest use of health resources and with the greatest intensity in the elderly population, as is the case of most of these patients and in the final stages of the disease [44]. In economic terms, it is estimated that at outpatient level, COPD represents an expense of 528 million euros a year. By patient and severity, a patient with serious COPD generated an expense of 3,335 euros a year, a patient with moderate COPD 2,275 euros/year and a patient with slight COPD 1,650 euros/year [44]. The proposed investigation would provide better knowledge on achieving the system’s sustainability through rational use of all available resources, and a health care that goes from primary care and specialised attention to home and palliative care.

A very significant goal of this study is to find out and encourage communication with patients, as well as their participation and collaboration in decision-taking. Involving patients in decision-taking is not only a right but also improves therapeutic results by avoiding treatment considered to be disproportionate and improving patients’ quality of life [45–47].

COPD has a major impact on quality of life due to dyspnoea, respiratory insufficiency, exacerbations and hospitalisations, leading to a reduction in productive activities, social relationships, significant dependence and social isolation. Our study seeks to contribute new knowledge regarding these patients’ needs and possible strategies to address them. In particular, the aim is to explore the use of opioids in these patients’ dyspnoea [48].

In conclusion, the study of advanced COPD’s natural background can help us to better understand what are the real assistance and palliative needs of these patients at end of life, with the aim of more efficiently organising their care at end of life.

### Limitations of the study

Our study has some possible limitations in design as regards the inclusion of participants and the parameters evaluated.

Firstly, the selection of patients mainly on the reference Neumology Services and the sample obtained may not be representative of the patients immobilised at home with a diagnosis of advanced (stage IV) COPD. It could therefore be necessary to use a recruitment strategy that also takes into account primary care doctors to access most of the patients in the area with these characteristics.

Secondly, as regards the variables evaluated, the registration dates of the diagnosis may not be totally exact, as they are based mainly on clinical registrations, and it is known to be a disease that is under-diagnosed and under-registered. However, most clinical information will be obtained directly from the patient or from the clinical record and the close, constant monitoring of these patients by the research team.

Thirdly, there may be losses in monitoring for reasons of institutionalisation, admittance or change of home. However, we think that the relationship of the ESAPD not only with the patient but with his or her close family environment, would allow this information to be recovered.

### Ethical aspects

Patients will be requested, freely and voluntarily, to give informed consent in writing if they are interested in taking part in the study, after reading the information sheet and clarifying any possible doubt they have about the study.

The project will be carried out in accordance with the basic principles of the Declaration of Helsinki and according to the legal regulations in effect (Royal Decree 223/2004) and having obtained the pertinent approvals from the reference CREC (Community of Madrid Primary Care and La Paz University Hospital, record PI2011) and the approval of its viability by the Central Committee on Research of the Primary Care Authorities (record 05/2012).

As regards data confidentiality, the study will comply with the terms laid down in the effective legislation: Organic Act 157/1999 of 13 December on Personal Data Protection (Official State Gazette number 298 of 14 December 1999), dissociating the data that could identify patients.
